# Effect of route of administration of dinoprost tromethamine on plasma profiles of 13,14-dihydro-15-keto-prostaglandin F_2α_ and progesterone in lactating Holstein cows

**DOI:** 10.3168/jdsc.2021-0142

**Published:** 2021-09-13

**Authors:** M.A. Mezera, M.R. Lauber, A.D. Beard, E.M. Cabrera, M.C. Wiltbank, P.M. Fricke

**Affiliations:** 1 Department of Animal and Dairy Sciences, University of Wisconsin-Madison, Madison 53706; 2 Endocrinology and Reproductive Physiology Program, University of Wisconsin-Madison, Madison 53706

## Abstract

Lutalyse HighCon (dinoprost tromethamine; Zoetis) has been approved for use both intramuscularly and subcutaneously in lactating dairy cows, although the effect of route of administration on circulating 13,14-dihydro-15-keto-prostaglandin F_2α_ (PGFM), the metabolite of PGF_2α_, has not been evaluated. Multiparous, lactating Holstein cows were submitted to an Ovsynch protocol in which the last GnRH treatment (G2) was designated as d 0. Cows were fitted with indwelling jugular catheters on d 6 and administered 25 mg of dinoprost tromethamine (2 mL of Lutalyse HighCon) on d 7 either subcutaneously in the neck (SC; n = 6) or intramuscularly in the semitendinosus muscle (IM; n = 6). Blood samples were collected every 15 min after treatment for 1.75 h, then every 2 h for 48 h, and at 60 and 72 h, with the last time point corresponding to when cows would have received timed AI at 72 h within an Ovsynch protocol. Circulating PGFM concentrations were greater for SC than for IM cows from 15 to 90 min after treatment, which resulted in a greater area under the PGFM curve during the first 90 min after treatment (means ± SEM; 1,664 ± 129 pg·h/mL vs. 1,146 ± 177 pg·h/mL for SC vs. IM cows, respectively). This resulted in complete luteolysis in all but one cow in the SC treatment at 56 h, when GnRH would have been administered if dinoprost tromethamine had been administered as part of an Ovsynch protocol for timed AI. For cows that underwent complete luteal regression, circulating P4 did not differ between treatments at any time point. Thus, although SC cows had increased circulating PGFM 15 to 90 min after treatment, there was no difference in circulating P4 during induced luteolysis based on route of dinoprost tromethamine administration.

## Highlights

• Effect of IM versus SC treatment with dinoprost tromethamine on PGFM is unknown.
• Circulating PGFM was greater for SC than IM cows 15 to 90 min after treatment.
• Circulating P4 did not differ between IM or SC cows with complete luteolysis.
• IM or SC treatment with dinoprost tromethamine can effectively induce luteolysis.

## Graphical Abstract

**Figure:**
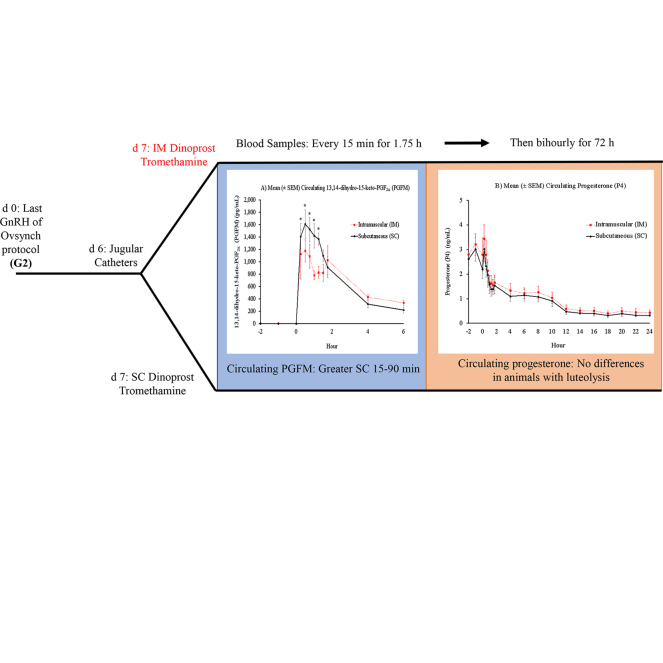
**Summary:** Our objective was to determine the effect of route of administration of dinoprost tromethamine 7 d after treatment with GnRH on circulating 13,14-dihydro-15-keto-prostaglandin F2α (PGFM) and progesterone (P4) concentrations in lactating dairy cows. Multiparous Holstein cows fitted with indwelling jugular catheters were randomized 7 d after the last GnRH of an Ovsynch protocol (G2, d 0) to receive 25 mg of dinoprost tromethamine either intramuscularly (IM) or subcutaneously (SC). The SC cows had greater circulating PGFM concentrations 15 to 90 min after treatment than the IM cows; however, circulating P4 concentrations during induced luteolysis did not differ at any time based on route of administration.

Prostaglandin F_2α_ is the luteolytic agent in ruminants (McCracken et al., 1972), and treatment with exogenous PGF_2α_ analogs such as dinoprost tromethamine is used to induce luteal regression in synchronization protocols in dairy cows (Wiltbank and Pursley, 2014). Dinoprost tromethamine is labeled for and is most commonly administered intramuscularly (**IM**), but it can induce luteolysis via several routes of administration, including cervical subcutaneous (**SC**) administration (Chebel et al., 2007), administration into the ischiorectal fossa (Colazo et al., 2002), intravaginal administration (Masello et al., 2020), and intrauterine administration (Ochoa et al., 2018). The effect of route of administration of dinoprost tromethamine [Lutalyse HighCon, 25 mg (2 mL); Zoetis] on circulating 13,14-dihydro-15-keto-prostaglandin F_2α_ (**PGFM**) concentrations in lactating dairy cows has not been well characterized.

Our objective was to determine the effect of route of administration with dinoprost tromethamine 7 d after the last GnRH treatment of an Ovsynch protocol (**G2**) on circulating PGFM concentrations, which is reflective of circulating PGF_2α_ concentrations (Samuelsson et al., 1975; Kindahl et al., 1976). In particular, this study focused on differences in SC and IM PGF_2α_ administration because dinoprost tromethamine under the brand name Lutalyse HighCon (Zoetis) is labeled for use both IM and SC for induction of luteolysis in the United States (US FDA, 2015). We hypothesized that route of administration would not affect rate of decrease in circulating progesterone (**P4**) concentrations in lactating dairy cows, as reported by Chebel et al. (2007). It is unclear, however, how route of administration might affect circulating PGFM profiles because similar rates of absorption for SC and IM administration were reported for some applications, whereas others reported slower release in SC administration, with individual differences in capillary density at the site of administration affecting overall rate of absorption (Prettyman, 2005).

All animal handling and experimental procedures were approved by the Animal Care and Use Committee of the College of Agriculture and Life Sciences at University of Wisconsin-Madison. Lactating, multiparous Holstein cows were submitted to an Ovsynch protocol as reported by Pursley et al. (1995) and modified by Brusveen et al. (2009), with G2 set as d 0. All cows were housed in a tiestall barn, fed a standard herd diet, and milked twice daily. On d 6, all cows were fitted with an indwelling jugular catheter as described elsewhere (Mezera et al., 2019) and randomized to 1 of 2 treatments on the morning of d 7 after collection of baseline blood samples 24, 2, 1, and 0 h before treatment. Cows were randomized to the following 2 treatments: (1) 25 mg (2 mL) of dinoprost tromethamine (Lutalyse HighCon; Zoetis) administered intramuscularly in the semitendinosus muscle (IM; n = 6), or (2) administered subcutaneously in the neck contralateral to the jugular catheter (SC; n = 6).

After administration of dinoprost tromethamine, blood samples were collected from the jugular vein every 15 min for 1.75 h. After that point, blood was collected every 2 h for an additional 46 h, as well as at 60 and 72 h after treatment. All blood samples were collected into 10-mL Vacutainers containing K_2_ EDTA (368589, Becton, Dickinson, and Co.). Plasma was collected and stored at −20°C until utilized for hormone analysis.

Concentrations of PGFM were evaluated using an ELISA based on the method reported by Ginther et al. (2010) with minor modifications reported by Mezera et al. (2019). The intra- and interassay coefficients of variation (CV) were 8 and 14%, respectively, and sensitivity was 5.2 pg/mL. Progesterone concentrations were evaluated using a commercial kit (CT Progesterone; 07-270105, MP Biomedicals LLC), following the manufacturer's instructions. The intra- and interassay CV were 5 and 2%, respectively, and sensitivity was 0.23 ng/mL. Results from hormonal analyses were analyzed in SAS 9.4 (SAS Institute Inc.) using the Proc Mixed procedure. A significant difference in the fixed effect was considered when *P* < 0.05.

Hormone profiles from both treatments were generated. For all cows, an increase in circulating PGFM occurred, with no difference in timing of the peak concentration between treatments (IM: 42 ± 16 min; SC: 50 ± 6 min; *P* = 0.67). In 4 of 6 IM cows and 3 of 6 SC cows, the initial peak in PGFM concentration was followed by a second, typically smaller peak in circulating PGFM concentrations (Figure 1: IM profiles, Figure 2: SC profiles). This secondary increase was particularly apparent in IM treatment cow 6645, where the secondary increase was nearly as large as the initial increase in PGFM concentrations. In aggregate, circulating PGFM concentrations were greater (*P* < 0.05) for SC cows than for IM cows from 15 to 90 min after treatment (Figure 3A), resulting in a greater (*P* = 0.04) area under the curve during the first 90 min for SC administration (1,664 ± 129 pg·h/mL vs. 1,146 ± 177 pg·h/mL for SC vs. IM cows, respectively). After 90 min, there was no difference in circulating PGFM concentrations at any time point (*P* > 0.05 at all points).

**Figure 1:**
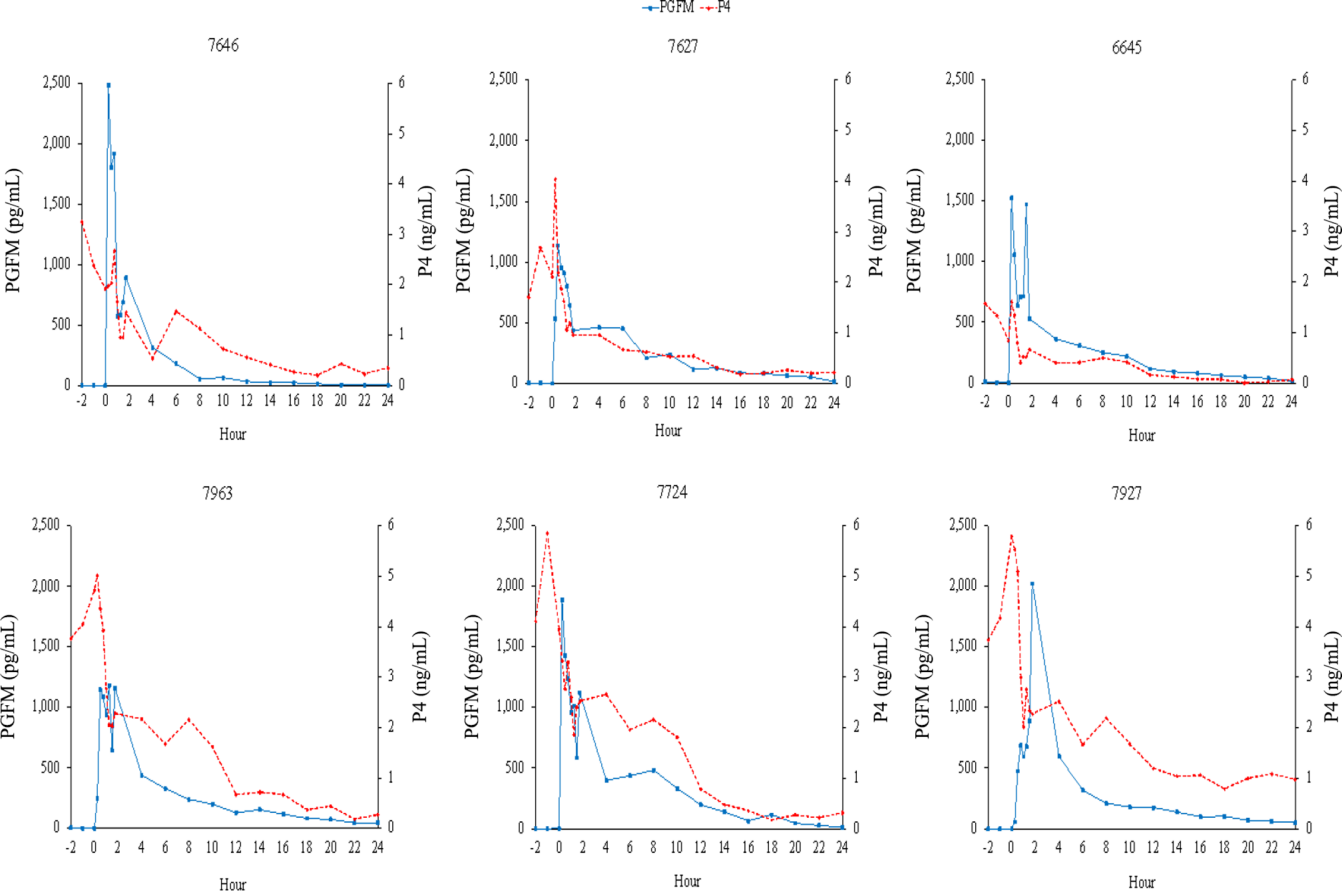
13,14-Dihydro-15-keto-prostaglandin F_2α_ (PGFM) and progesterone (P4) concentrations (pg/mL and ng/mL, respectively) for individual cows from −2 h to 24 h relative to intramuscular treatment with dinoprost tromethamine (0 h; n = 6).

**Figure 2:**
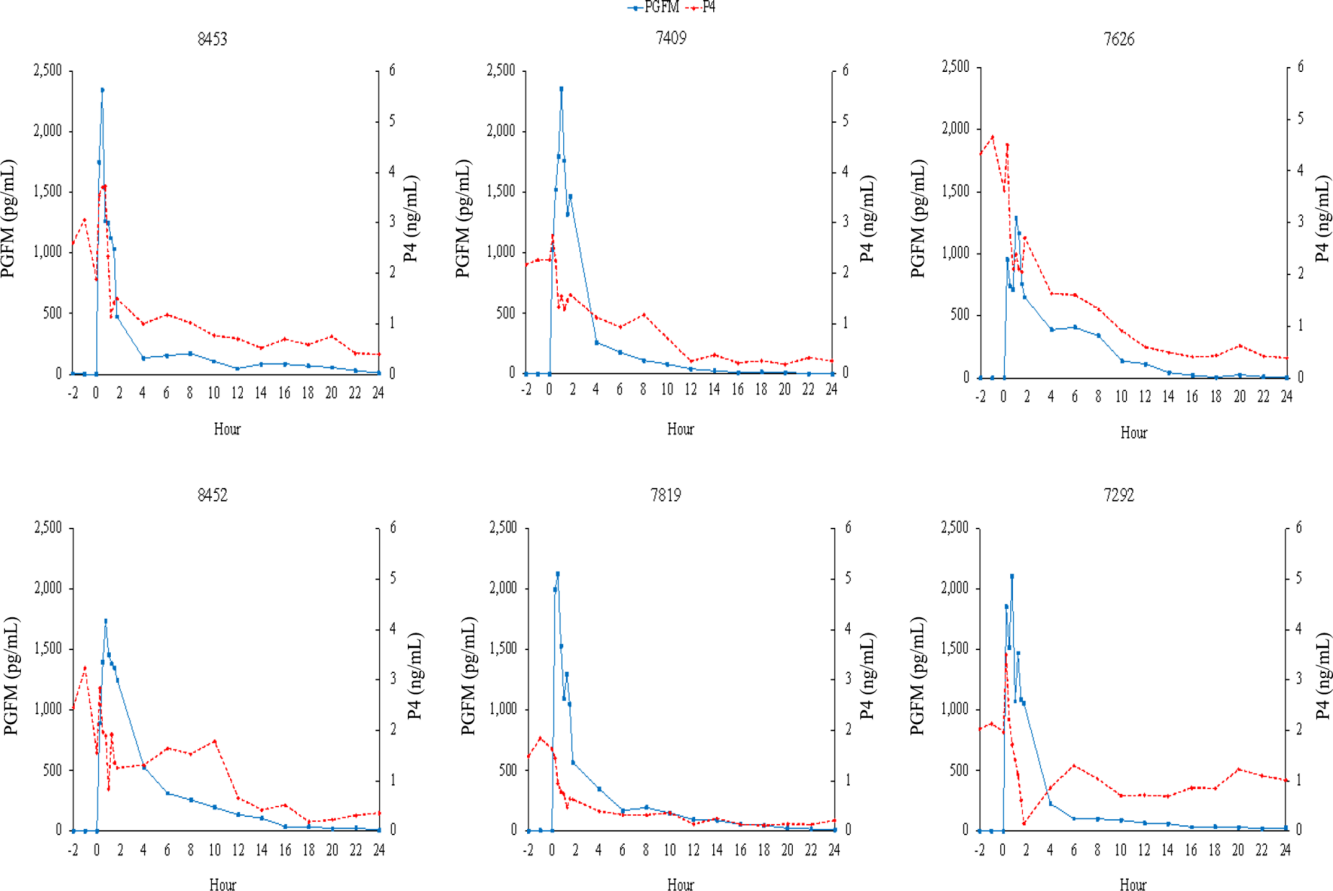
13,14-Dihydro-15-keto-prostaglandin F_2α_ (PGFM) and progesterone (P4) concentrations (pg/mL and ng/mL, respectively) for individual cows from −2 h to 24 h relative to subcutaneous treatment with dinoprost tromethamine (0 h; n = 6).

**Figure 3:**
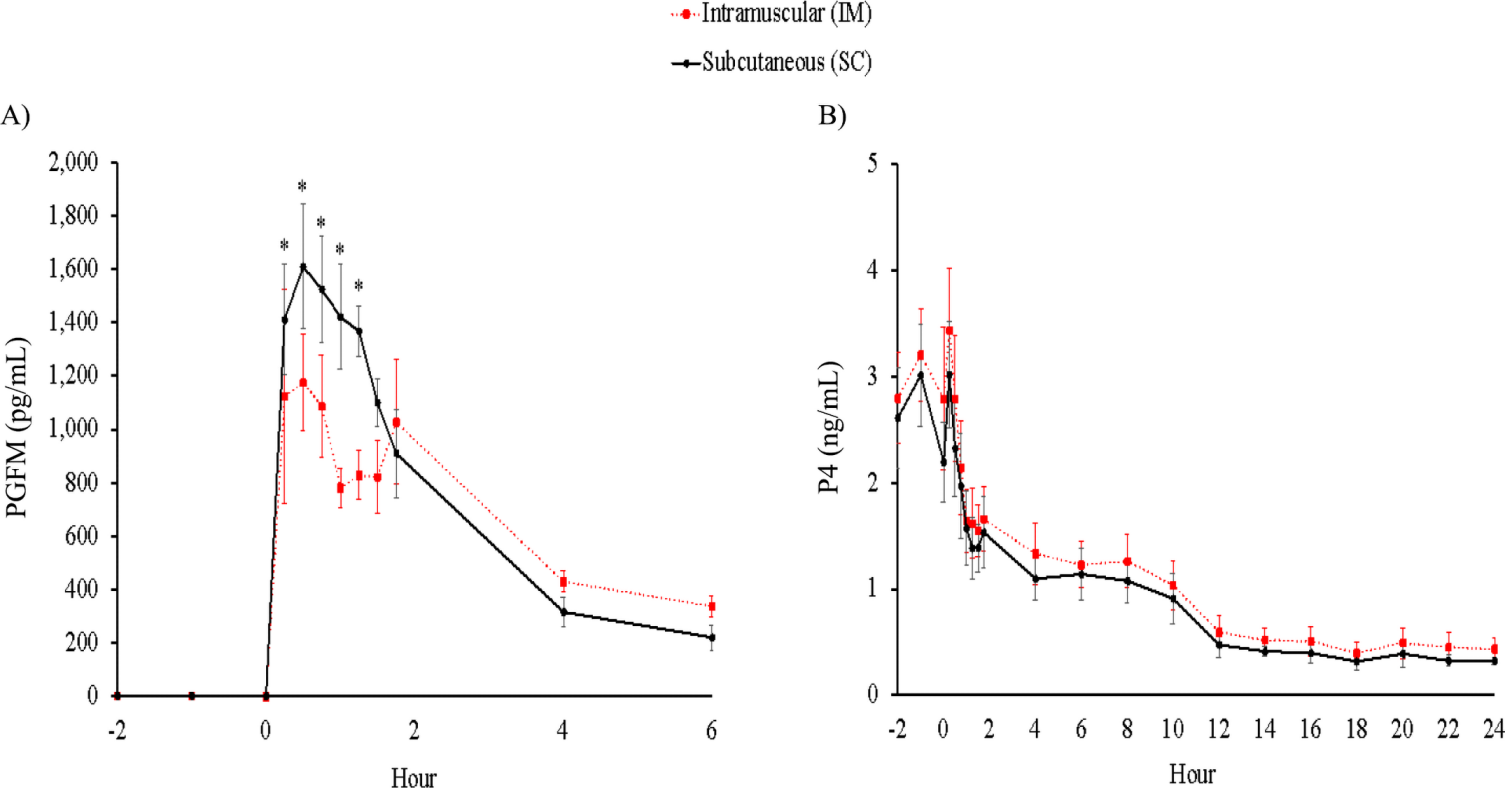
(A) Mean (±SEM) circulating 13,14-dihydro-15-keto-prostaglandin F_2α_ (PGFM) −2 to 6 h relative to treatment (0 h) with dinoprost tromethamine intramuscularly (IM; n = 6) or subcutaneously (SC; n = 6). (B) Mean (±SEM) circulating progesterone (P4) −2 to 24 h relative to treatment (0 h) with dinoprost tromethamine IM (n = 6) or SC (n = 5). Cow 7292 (SC treatment) was removed from the circulating P4 analysis due to failure to undergo complete luteolysis by 56 h (P4 <0.5 ng/mL) with P4 of 1.46 ng/mL.

In 5 of 6 SC cows and 4 of 6 IM cows, treatment with dinoprost tromethamine was followed by a transient increase in circulating P4 15 min after treatment, followed by a gradual decrease in circulating P4 concentrations in all cows (Figure 1: IM profiles, Figure 2: SC profiles). This resulted in complete luteal regression (P4 <0.5 ng/mL) in 5 of 6 cows in the SC treatment and 6 of 6 cows in the IM treatment at 56 h, the time at which GnRH would be administered during an Ovsynch protocol. Luteal regression was incomplete in the SC treatment for cow 7292, which had a circulating P4 concentration of 1.46 ng/mL at 56 h and 1.76 ng/mL at 72 h. Due to failure to undergo complete regression, this cow was eliminated from comparisons of circulating P4 concentrations. A comparison of circulating P4 concentrations found no difference at any time point due to route of administration of dinoprost tromethamine (first 24 h of P4 between treatments: Figure 3B; *P* > 0.05, all time points).

From a practical standpoint, although this study indicated that there are differences between treatments in average circulating PGFM concentrations, the lack of a difference in circulating P4 concentrations after PGF_2α_ administration in the present study, as well as in larger studies (Chebel et al., 2007), indicates that both methods of administration are acceptable for induction of luteolysis in lactating dairy cows.

The average differences in PGFM concentrations and similarities in P4 concentrations are important for management decisions when implementing hormonal synchronization protocols, but closer inspection of individual cows yields intriguing possibilities for luteal physiology. For cows in both treatments, there was a secondary increase in circulating PGFM concentrations after the initial increase associated with dinoprost tromethamine treatment, with this pattern being observed in a total of 7 cows. This has been observed in another study in which cows were treated with 25 mg of dinoprost tromethamine IM (Greco et al., 2015). The secondary increase in PGFM concentrations leads to speculation of 2 potential hypotheses based on our current understanding of PGF_2α_ signaling. The first is based on the ability of PGF_2α_ to induce vasoconstriction (Morimoto et al., 1990). In this hypothesis, the initial introduction of PGF_2α_ causes vasoconstriction, preventing efficient uptake of additional PGF_2α_ until concentrations decrease enough to allow relaxation of vascular tissue and subsequent release of more dinoprost tromethamine and a secondary increase in PGF_2α_. The second hypothesis is based on auto-amplification of PGF_2α_ secretion, which has been observed with cloprostenol, which induces additional PGF_2α_ secretion from the uterus (Duong et al., 2012). In this scenario, exogenous PGF_2α_ administration could act like natural luteolytic PGF_2α_ pulses to induce PGF_2α_ release from the uterus, thereby leading to the increase in circulating PGFM.

Although both hypotheses are speculative, understanding why some cows have a secondary increase in circulating PGFM concentrations and what controls the magnitude of this secondary increase could lead to strategies to increase efficacy of exogenous PGF_2α_ to induce luteal regression, because multiple pulses of PGF_2α_ increase the efficacy of luteal regression (Ginther et al., 2009). This mechanism would increase luteal regression and potentially overall synchrony to hormonal synchronization protocols because cows that fail to regress their CL in response to exogenous PGF_2α_ or that undergo incomplete luteal regression have low fertility to timed AI (Carvalho et al., 2018).

In summary, although route of administration did not affect average circulating P4 concentrations after dinoprost tromethamine treatment, we observed differences in circulating PGFM concentrations, with cows receiving SC administration having greater circulating PGFM concentrations 15 to 90 min after treatment compared with cows receiving IM administration. This indicates that both routes of PGF_2α_ administration are effective for use in synchronization protocols in lactating cows. Inspection of individual cows revealed the presence of a secondary increase in PGFM concentrations after dinoprost tromethamine treatment in about half of the cows, regardless of route of administration, with a high degree of variation among cows in amplitude of this secondary PGFM increase. These observations led to speculation on the consequences of this additional increase, with a better understanding of this process potentially contributing to a fuller understanding of luteal physiology and a possible opportunity to increase the efficacy of treatment with exogenous PGF_2α_ on luteal regression in lactating dairy cows.
